# Microglia Phenotype and Intracerebral Hemorrhage: A Balance of Yin and Yang

**DOI:** 10.3389/fncel.2021.765205

**Published:** 2021-10-13

**Authors:** Rentang Bi, Zhi Fang, Mingfeng You, Quanwei He, Bo Hu

**Affiliations:** Department of Neurology, Union Hospital, Tongji Medical College, Huazhong University of Science and Technology, Wuhan, China

**Keywords:** intracerebral hemorrhage, microglia, neuroinflammation, neuroprotective, stroke

## Abstract

Intracerebral hemorrhage (ICH) features extremely high rates of morbidity and mortality, with no specific and effective therapy. And local inflammation caused by the over-activated immune cells seriously damages the recovery of neurological function after ICH. Fortunately, immune intervention to microglia has provided new methods and ideas for ICH treatment. Microglia, as the resident immune cells in the brain, play vital roles in both tissue damage and repair processes after ICH. The perihematomal activated microglia not only arouse acute inflammatory responses, oxidative stress, excitotoxicity, and cytotoxicity to cause neuron death, but also show another phenotype that inhibit inflammation, clear hematoma and promote tissue regeneration. The proportion of microglia phenotypes determines the progression of brain tissue damage or repair after ICH. Therefore, microglia may be a promising and imperative therapeutic target for ICH. In this review, we discuss the dual functions of microglia in the brain after an ICH from immunological perspective, elaborate on the activation mechanism of perihematomal microglia, and summarize related therapeutic drugs researches.

## Introduction

Intracerebral hemorrhage (ICH) has become one of the most common and lethal diseases in the last decades (Zhou M. et al., 2019). It affects more than 2 million patients worldwide every year, with the majority in developing countries (Cordonnier et al., 2018; Zhu et al., 2019). ICH represents 10–25% of all strokes but leads to more than 50% of the deaths (Lan et al., 2017b; Cordonnier et al., 2018). 43–51% of patients with ICH die within 30 days, and only 12–39% of survivors keep living independently which imposes an enormous burden upon healthcare systems (Zhou et al., 2014; An et al., 2017). Neither internal medical managements, including hemostasis and intensive blood pressure-reduction, nor surgery methods as hematoma evacuation, has been testified efficacious by clinical randomized controlled trials (Mayer et al., 2008; Mendelow et al., 2013; Hemphill et al., 2015; Baharoglu et al., 2016; Morotti et al., 2017; Cordonnier et al., 2018). However, inspiringly, immune intervention promises a specific therapy strategy when neurologists shift attention to ICH secondary injury. Lately, fingolimod has been demonstrated signally improved neurological functional recovery in patients with ICH by means of regulating immunocytes number and activity (Fu et al., 2014; Li Y.-J. et al., 2015).

Microglia, as the resident immunocyte accounting for 5–10% of all human brain cells (Ma et al., 2017; Liu et al., 2021), take the lead in both tissue damage and repair processes after ICH. The perihematomal activated microglia not only arouse acute inflammatory responses, oxidative stress, excitotoxicity, and cytotoxicity to damage neurovascular unit (M1 phenotype), but also transform the phenotype to inhibit inflammation, clear hematoma, and promote tissue regeneration (M2 phenotype). M1 and M2 microglial phenotypes play opposite functions, but they are actually complementary, interconnected, and can be transformed into each other, work coordinately and even interdependently (Hu et al., 2015; Orihuela et al., 2016), just like yin and yang in ancient Chinese philosophy. Their balance directly determines which way the pathophysiology goes towards, brain tissue repair or excessive damage. Thus, microglia may be a promising and imperative therapeutic target for ICH.

In this review, we describe the dualistic roles of microglia in ICH from an immunological perspective, expound on the detailed mechanism of perihematomal microglial activation and polarization, and summarize the related therapeutic researches.

## Microglia

German neuropathologist Franz Nissl firstly discovered microglia with platinum stain in 1899 and called it “Staebchenzellen”. Then, Spanish neurohistologist Del Rio-Hortega coined the term “microglia” in 1919 and described in detail its superior ability of rapid proliferation, migration, and phagocytosis, which laid the groundwork for follow-up studies (Ginhoux and Prinz, 2015; ElAli and Rivest, 2016; Smolders et al., 2019).

After a century of exploration, microglia are customarily regarded as the macrophage in the brain due to the similarity in morphology, functions, and biomarkers (Nayak et al., 2014; Ginhoux and Prinz, 2015). Microglia can be identified with classical macrophage markers, such as ionized calcium binding adapter molecule1 (Iba1), surface glycoprotein F4/80, integrin CD11b, and the epitope of keratan sulfate 5D4 (Nayak et al., 2014; Dudvarski Stankovic et al., 2016; Lan et al., 2017b). However, microglia have been demonstrated to possess different embryological origin and transcriptional profile from that of macrophage, which suggest the functions of microglia and microphage are not identical. Microglia are recognized as Tmem119-positive and CD45-low, while macrophages are Tmem119-negetive and CD45-high (Li Q. et al., 2018).

Activated microglia have been found to differentiate into two broad subtypes with distinct cellular makers and biological functions (Sica and Mantovani, 2012; Zhao H. et al., 2015; Dudvarski Stankovic et al., 2016; Lan et al., 2017b; Ma et al., 2017; Li Q. et al., 2018; Tschoe et al., 2020). According to the M1/M2 dichotomy proposed by Mills in 2000, activated microglia are categorized into pro-inflammatory M1 phenotype (classical activation) and anti-inflammatory M2 phenotype (alternative activation). The process that resting microglia differentiate into M1/M2 phenotype is referred to as polarization. Recently, M2 microglia are alternatively divided into M2a/M2b/M2c subtypes. Classical inflammatory factors such as IL-1β, IL-6, and Tumor necrosis factor-α (TNF-α) were used as the main markers of M1 microglia, while M2a microglia markers are represented by anti-inflammatory factors IL-4, IL-10, scavenger receptor CD36, and mannose receptor CD206, M2b microglia express major histocompatibility complex II (MCH-II), CD86, IL-10, and M2c microglia express phagocytic receptor CD163, insulin-like growth factor 1 (IGF-1), brain-derived neurotrophic factor (BDNF). Different markers of microglia phenotypes show different roles that they play after ICH. The particular information on microglia subtypes is summarized in Table 1 (Lan et al., 2017b; Ma et al., 2017; Tschoe et al., 2020; Liu et al., 2021).

**Table 1:** Particular information on microglia subtypes.

Phenotype	Polarization agents	Makers	Roles
M1	LPS, IFN-γ, TNF-α, IL-1β, IL-17	IL-1β, IL-6, IL-12, IL-23	pro-inflammation
		TNF-α	pro-inflammation
		iNOS	oxidative damage
		MHC-II	antigen presentation
		CCL2, CCL5, CCL20	chemokine
		CXCL10	chemokine
		MMP2, MMP9	matrix decomposition
		CD16, CD32	phagocytosis, chemotaxis
M2			
M2a	IL-4, IL-13	IL-4, IL-10	anti-inflammation
		TGF-β	anti-inflammation
		CD36	phagocytosis
		CD206	phagocytosis
		CCL22	chemokine
		Arg-1	tissue regeneration
		Ym-1	stabilizing extracellular matrix
		Fizz1	tissue regeneration
M2b	TLRs agonist, IL-1R ligands, Fc receptors	MCH-II	pro-inflammation
		CD86	pro-inflammation
		IL-1RA	anti-inflammation
		IL-10	anti-inflammation
M2c	IL-10, TGF-β, glucocorticoid	CD163	phagocytosis
		IGF-1	tissue regeneration
		NGF	tissue regeneration
		BDNF	tissue regeneration
		NT3, NT4/5	tissue regeneration
		Arg-1	tissue regeneration
		YM-1	stabilize extracellular matrix
		Fizz1	tissue regeneration

*LPS, lipopolysaccharide; IFN-γ, interferon γ; TNF-α-II, tumor necrosis factor α; iNOS, inducible nitric oxide synthase; MHC-II, major histocompatibility complex II; MMP, matrix metalloproteinase; Arg-1, arginine 1; Ym-1, chitinase 3-like 3; IGF-1, insulin-like growth factor 1; NGF, nerve growth factor; BDNF, brain-derived neurotrophic factor; NT3, neurotrophin 3; NT4/5, neurotrophin 4/5; FIZZ1, resistin-like-α*.

## Spatiotemporal Pattern of Microglial Activation After ICH

As the immune monitor in the brain, microglia become activated immediately after ICH, make morphological changes from a highly ramified phenotype to a rod, spherical, and finally an amoeba shape with contracting, thickening, and largening (more than 7.5 μm in diameter; Walker et al., 2014; Yang S. S. et al., 2016; Shtaya et al., 2019; Wei et al., 2020).

Spatially, microglia usually show different activation levels, morphologies (ameboid, branched, or intermediate), and directivities in different distances from the hematoma (Wang G. et al., 2013; Yang S. S. et al., 2016). Amoeba microglia mainly appear in close proximity to the hematoma, and partial microglia are found activated away from the hematoma, such as the ipsilateral cerebral cortex, corpus callosum, and hippocampus.

**Figure 1:**
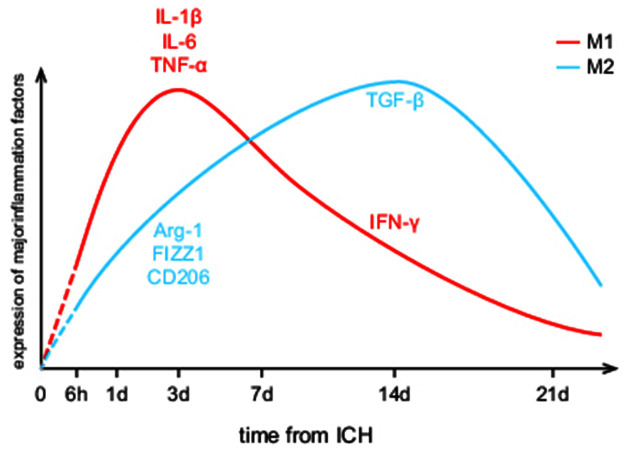
Dynamic changes of M1/M2 microglial activation levels after intracerebral hemorrhage (ICH). It provides a visual expression that M1/M2 microglia take on different activation characteristics. The red curve represents M1 microglia while the blue curve represents M2 microglia. Yet, the referenced researches about microglial spatiotemporal features are all animal experiments, leaving the human brain as an unknown area.

In the time course, microglia activation begins within 1–4 h, peaks in 1–3 days, declines at day 7, and returns to physiological level in 3–4 weeks after ICH (Zhou et al., 2014; Wan et al., 2016; Zhu et al., 2019). As shown in Figure 1, both M1 and M2 phenotypes of microglia are presented in the perihematomal area throughout the course of the disease, while the M1/M2 proportion is continually changing. It stays in an M1-dominated state for a week after ICH and deflects to an M2 preponderance within 1–2 weeks (Wan et al., 2016). In animal models, M1 makers including IL-1β, IL-6, TNF-α, including inducible nitric oxide synthase (iNOS) increase dramatically within 3 days after ICH, while interferon-γ (IFN-γ) mostly increase in the later phase. The levels of M2 makers like Arginase-1 (Arg-1), resistin-like-α (Fizz1), CD206 go up gradually within 1 week and decline in 7–14 days except for transforming growth factor-β (TGF-β), which remains relatively high at days 14 (Zhao H. et al., 2015; Dang et al., 2017; Lan et al., 2017b; Taylor et al., 2017).

Notably, despite the time point is different, almost all microglial markers increase, which makes it difficult to faultlessly describe the dynamic phenotypic changes. With regard to this fact, it is better to evaluate microglial activation with as many makers as possible at present.

## Functions of Activated Microglia After ICH

**Figure 2:**
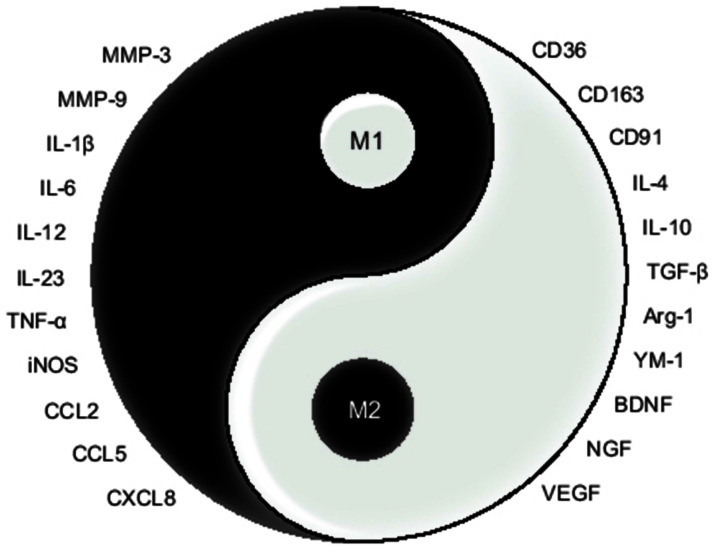
Sketch map for the opposite function of M1/M2 microglia. In this Tai Chi-diagram, the half-filled-out symbols with left half black represent M1 microglia, with the expression of MMPs, pro-inflammatory cytokine, and chemokine. And the right half white represents M2 microglia, with the expression of phagocytic receptors, anti-inflammatory cytokine, and growth factors.

After ICH, blood swarms into the brain parenchyma causing an expanding hematoma which leads to immediate neurological impairment and microglial activation. Respectively, M1 microglia are commonly considered as the deleterious phenotype, and M2 microglia as the beneficial one (Xi et al., 2014; Zhou et al., 2014), as shown in Figure 2. Microglia possess phenotypic and functional plasticity. Promoting M1-M2 phenotypic transformation has become the mainstream strategy of microglial intervention in ICH treatment.

### M1 Microglia

M1 microglia secrete a large number of inflammatory factors, proteases, chemokines, prostaglandins, and other toxic substances. Since multiple damage-inducing factors overlap, brain cells die in various forms such as apoptosis, necrosis, pyroptosis, ferroptosis, which leads to the irreversible destruction of brain structure (Xi et al., 2014; Zhou et al., 2014).

In brain parenchyma, M1 microglia are the major source of inflammatory mediators, such as IL-1β, IL-6, IL-12, IL-23, and TNF-α (Jiang et al., 2020). Although inflammation is essential for innate immunity, it is the chief culprit to the sustained neurological deterioration in a sterile environment (Zhu et al., 2019). While inflammatory cytokines diffuse, functional neurons and neuroglia quickly die under the stress condition (Shen et al., 2017). The diffused inflammatory cytokines also promote polarization of surrounding microglia towards the M1 phenotype, cause the inflammatory region to expand, which forms a vicious circle. In patients with ICH, the levels of IL-1β, TNF-α, and IL-6 in plasma and brain tissues are significantly increased within 1–3 days, and the increasing degree is related to 90-days poor prognosis (Jiang et al., 2020). During pathological processes, oxidative stress and inflammation mutually reinforcing, which is no exception in ICH (Hu et al., 2016; Yao et al., 2021). M1 microglia express large amounts of peroxidases, iNOS, and reduced form of nicotinamide-adenine dinucleotide phosphate (NADPH) oxidase, which produce excessive free radicals and damage surrounding cells by attacking cellular membranes and DNA (Yang et al., 2013; Duan et al., 2016; Hu et al., 2016; Xiong et al., 2016). Moreover, M1 microglia contribute to the activation of matrix metalloproteinases (MMPs), including MMP2 and MMP9, which markedly destruct the blood-brain barrier (BBB) and cause severe vasogenic brain edema by degrading extracellular matrix constituents and attacking endothelial claudin-family tight junction proteins (Montaner et al., 2019). In ICH patients, increased MMP2/9 levels were independently associated with perihematomal edema volume (Li et al., 2013). In addition, M1 microglia also release chemokines including CXCL8, CCL2, and CCL5, which diffuse into peripheral blood through the ruptured blood vessel and attract peripheral leukocytes such as neutrophils, monocytes, and lymphocytes into brain parenchyma through disrupted BBB (Trettel et al., 2020). It was reported that chemokines concentrations in plasma were proportional to the infiltration degree of peripheral immunocytes in ICH patients (Guo et al., 2020). The infiltrated immunocytes not only express and secrete inflammatory factors and aggravate inflammatory response but also release toxic substances after their apoptosis (Lambertsen et al., 2019). In ICH patients, CCL2 concentrations in plasma within 24 h were associated with poor functional outcomes at day 7 after ICH (Hammond et al., 2014). Also, inhibiting CCL2 in animal models reduced brain edema and improved neural function (Yan et al., 2020).

Noticeably, there is an evident cooperativity effect on tissue damage induced by inflammatory cytokines, protease MMPs, and chemokines. Inflammatory cytokines not only attack vascular endothelial cells and tight junction proteins but also induce endothelial cells to secrete intercellular cell adhesion molecule-1 (ICAM-1), which promotes the adhesion and infiltration of peripheral leukocytes (Aslam et al., 2012). The direct damage on neurons induced by MMPs exacerbates inflammatory response, disrupts BBB to facilitate peripheral leukocytes infiltration (Kim et al., 2005). The infiltrated peripheral leukocytes secrete inflammatory factors and MMPs, which aggravates inflammatory response and BBB destruction in turn (Tschoe et al., 2020).

Although the treatments aiming at inflammatory cytokines are currently limited in animal experiments, TNF-α antibody has shown huge therapeutic potential by significantly reducing the number of perihematomal activated microglia and improving neurological outcomes in mouse stroke models (Mayne et al., 2001; Lei B. et al., 2013; Chen A.-Q. et al., 2019). Inhibition of TNF-α not only reduces the microglial activation/macrophage recruitment *via* decreasing cleaved caspase-3 level (Mayne et al., 2001; Lei B. et al., 2013; Chen A.-Q. et al., 2019) but also reduces the activation of TNF receptor 1 (TNFR1) on endothelium therefore reducing endothelium necroptosis and ameliorating disruption of BBB (Mayne et al., 2001; Lei B. et al., 2013; Chen A.-Q. et al., 2019). Predictably, inhibition of specific inflammatory factors is becoming the central theme of ICH therapeutic researches.

### M2 Microglia

M2 microglia primarily express anti-s and facilitate tissue regeneration (Lan et al., 2017b). Thereby, the injured brain acquires comprehensive and effective recovery. Due to the large amounts of anti-inflammatory cytokines and antioxidants, the inflammatory response and oxidative become diminished tardily (Zhu et al., 2019). More importantly, the anti-inflammatory factors promote surrounding microglia and other immune cells to transform into anti-inflammatory phenotype. It’s found that patients with higher TGF-β levels in plasma had a better prognosis at 90 days after ICH (Jiang et al., 2020).

At the same time, M2 microglia engulf the hematoma and cells debris, remove harmful substances and provide space for tissue regeneration. With the increase of the number of M2 microglia, the volume of the hematoma is eliminated promptly in 7–21 days after ICH. B-scavenger receptor CD36, one of the M2 microglial makers, is the main executive of microglial phagocytosis activity, which is obviously induced to upregulate by IL-10 (Fang et al., 2014; Yang et al., 2015; Li et al., 2021). In the mouse ICH model, CD36 knockout significantly inhibits hematoma absorption, and leads to the aggravation of neurological disorders (Fang et al., 2014). Instead, adoptive transferring CD36-positive microglia to CD36 knockout mice showed a significant improvement of neurological function after ICH (Yang et al., 2015). In fact, M2 microglia express CD163 and CD91 to absorb hemoglobin and heme, respectively (Dang et al., 2017; Garton et al., 2017). It should be noted that CD163 levels expressed by microglia may not be the only limiting factor in hematoma clearance. As a protective mechanism against severe hemolysis, the Haptoglobin (Hp) secreted by oligodendrocytes can capture free hemoglobin (Hb) to form a stable Hp-Hb complex, which is then englobed through CD163, thus reducing the toxicity of Hb. Similarly, hemopexin (Hx), secreted by neurons, binds with heme and is devoured *via* CD91 (Ma et al., 2016).

Particularly, M2 microglia are the drivers of brain tissue regeneration and remodeling. M2 microglia express various growth factors and trophic factors, such as insulin-like growth factors-1 (IGF-1), Brain-derived neurotrophic factor (BDNF), glial cell line-derived neurotrophic factor (GDNF), neurotrophin 3 (NT-3), NT-4/5, which could promote neurogenesis and neural circuit reframing (Xi et al., 2014; Ma et al., 2017). IGF-1 promotes the proliferation, migration, and differentiation of the neuro precursor cells in the subventricular zone, and facilitates the regenerated neurons’ functional integration into a new neural circuit (Thored et al., 2009). In a mouse ICH model, IGF-1 antibody promotes microglial M1 polarization, leading to more residual behavioral defects (Sun et al., 2020). BDNF and GDNF stimulate axon regeneration, which takes part in new neural connections (Madinier et al., 2009). The neurotrophic factors, including NT3 and NT4/5, are not only beneficial to the survival of residual neurons but also essential for the improvement and stability of the newborn neuron (Ma et al., 2017). During the remodeling of brain tissue, M2 microglia secrete clotting substance chitinase 3-like 3 (Ym-1) to prevent the degradation of extracellular matrix components (Girard et al., 2013). M2 marker Arg-1 not only converts arginine into polyamine which contributes to extracellular matrix subsidence but also competes with iNOS for reaction substrates to inhibit the excessive oxidative stress (Munder, 2009).

In general, M2 microglia resist inflammation and engulf hematomas to create a calm and stable microenvironment, which contributes to the neuro-angiogenesis and matrix deposition, and allows brain tissue to regain structure and function. Nevertheless, because of M1 microglia domination, only a third of new neurons survive inflammation in the acute phase. Therefore, promoting a beneficial microglial phenotypic transformation is a promising way in ICH treatment.

## Polarization Mechanism of Microglia After ICH

In order to regulate microglial polarization accurately and effectively, it is necessary to understand the mechanism of microglial polarization, including the source of extracellular stimuli and intracellular signaling pathways, which has been briefly summarized in Table 2.

**Table 2:** Signaling pathways of microglia polarization.

Microglia phenotype	Intracellular signal molecule	Extracellular agents	Effect molecules
M1	TLRs-NF-κB	Hb, hemin	NLRP3; IL-1β, IL-6, TNF-α
		fibrinogen	
		HMGB1, nucleic acids, heat shock protein	
		Prxs	
	MAPK-NF-κB	IL-1β, IL-6, TNF-α	
		thrombin	
		glucocorticoid	
	STAT1	IL-1β, IL-6, TNF-α
M2	PPAR/Nrf2	Peroxisome	Arg-1, IL-4, CD36, HO-1
	STAT4/6	IL-4, IL-10	Ym-1, Fizz1

### Extracellular Agents

**Figure 3:**
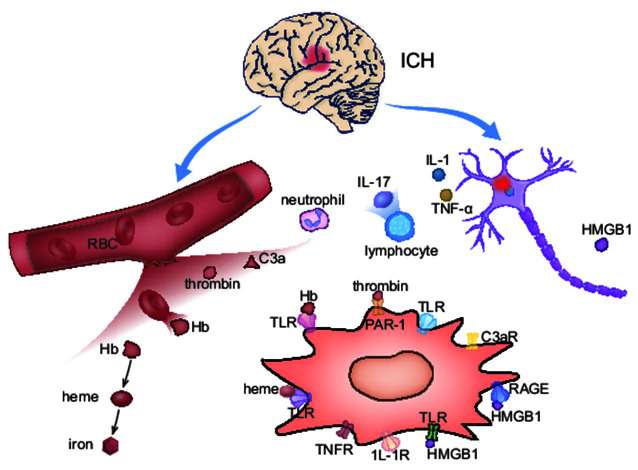
The activation mechanism of M1 microglia after ICH. In ICH acute phase, M1 microglia are activated on account of the blood composition and neuron primary damage. Meanwhile, microglia up-express corresponding receptors for activation.

After ICH, blood carrying red blood cells (RBCs) and plasma proteins including thrombin and fibrinogen infiltrate into the brain parenchyma, and trigger the initiation of early cellular and molecular pathological processes. Hematoma not only contains the agents that directly activate microglia but also promote microglial M1-polarization indirectly through tissue damage. Figure 3 provides an overview of M1-polarization.

Because of energy exhaustion and cytotoxicity, RBCs in the hematoma begin to lyse within 1 day and continue for weeks after ICH (Righy et al., 2016). The damaged RBCs release Hb, peroxiredoxins (Prxs), and Carbonic Anhydrase-1 (CA-1), which induce microglia differentiating into M1-phenotype (Guo et al., 2012; Liu et al., 2016; Bian et al., 2020). Hb and the decomposed product hemin can directly promote microglial M1-polarization through Toll-like receptors (TLRs; Lin et al., 2012; Wang et al., 2014). Therefore, clearing hematoma is of importance in reducing brain damage. Since no reliable clinical benefits are provided from surgical hematoma removal at present, promoting hematoma devouring by microglia is of great significance.

During the formation of hematoma, thrombin and complements are produced in the brain, which are also important factors for M1-polarization. Thrombin, a serine protease that promotes blood clotting, is detected in the brain within 1 h after ICH (Zhu et al., 2019). Thrombin directly activates M1 microglia by binding to the proteinase-activated receptor-1 (PAR-1; Wan et al., 2016). In mouse models, delayed administration of thrombin inhibitor hirudin in 7–28 days after ICH significantly reduced the number of pro-inflammatory microglia (Li et al., 2019). However, thrombin regulation is difficult to apply to clinical therapy because of its two-sided effects. Though the inhibition of thrombin shows a beneficial effect in inflammation reduction, a suitable thrombin concentration is necessary for helping stop hemorrhage and protect neurons. Complements, anaphylatoxins, are activated within 24 h through various proximal cascaded pathways (Ducruet et al., 2009; Yuan et al., 2017). Complement composition C3a activates microglia cells by binding to the specific receptor C3aR. Membrane attack complex (MAC), the end product of complement cascade, attacks cell membrane, and leads to erythrocyte lysis and neuronal death, which indirectly exacerbates microglial M1-polarization. In animal models, complement inhibitor N-acetyl heparin inhibits microglia activation and ameliorates neurological deficits (Wang M. et al., 2019).

Besides, brain tissue primary damage also contributes to microglial polarization (Zhang et al., 2017). Neurons and astroglia around the hematoma express inflammatory factors such as IL-15 and IL-17, playing a vital role in M1 polarization (Yu et al., 2016; Shi et al., 2018, 2020). Likewise, damaged neurons and glia release damage-associated molecular patterns (DAMPs), including high mobility group protein-1 (HMGB1), heat shock proteins, and extracellular matrix fragments (Mracsko and Veltkamp, 2014; Bobinger et al., 2018). HMGB1 is a non-chromosome-related protein widely expressed in the nucleus of all eukaryotic cells (Mu et al., 2018). Under physiological conditions, HMGB1 helps stabilize chromosomes and regulate the transcription of many survival-based genes, but once it is dissociated from the nucleus and released outside the cell, HMGB1 becomes a powerful inflammatory mediator that promotes microglial M1 polarization by binding to TLRs on microglia (Ohnishi et al., 2011; Wang D. et al., 2017). In rodent ICH models, glycyrrhizin attenuates intracerebral hemorrhage-induced injury in a concentration-dependent manner *via* inhibiting HMGB1 (Ohnishi et al., 2011; Mu et al., 2018). HMGB1 inhibitor Ethyl-pyruvate significantly reduced microglia activation and inflammatory factors levels *via* inhibiting nuclear factor kappa B (NF-κB) DNA binding activity (Su et al., 2013).

In the later phase of ICH, an anti-inflammatory pathway, enlisting native microglia, occurs alongside neuroinflammation (Shtaya et al., 2021). Anti-inflammatory factors such as IL-4, IL-33, IL-10, TGF-β increase distinctly around the hematoma, which are mostly released by macrophages, mature lymphocytes, and mast cells (Taylor et al., 2017; Zhou et al., 2017; Chen Z. et al., 2019). The immune microenvironment changes shift microglial polarization from M1 to M2. Intraventricular injection of IL-4 in mice increases the proportion of M2 microglia and accelerates the recovery of neurological function after ICH (Yang J. et al., 2016). Some other molecular targets have also been recently identified up-regulated on microglia during M2-polarization after ICH, including Dopamine D1 receptor (DRD1), Cannabinoid receptor-2 (CB2R), Melanocortin receptor 4 (MC4R), and especially sphingosine-1-phosphate receptor (S1PR; Xu et al., 2013; Li L. et al., 2015; Zhang et al., 2015).

### Intracellular Signal Transduction

To recognize extracellular agents and transduce extracellular signals, microglia express various membrane receptors, nuclear receptors, and executive proteins to play roles in morphological and functional changes such as secretion, phagocytosis, and movement. Understanding microglial signal transduction is beneficial to the exploration of clinical targets. Here, we briefly introduce several important receptors and signaling molecules.

#### TLRs-NF-κB

TLR is a type I transmembrane protein that plays an important role in the innate immune and inflammatory response (Alvarado and Lathia, 2016). So far, 10 functional TLRs have been found in humans, and microglia mainly express TLR4, TLR2, and heterodimer TLR2/4 (Fang et al., 2013; Hayward and Lee, 2014; Wang et al., 2014). Hb, hemin, fibrinogen, HMGB1, heat shock protein, Prxs, and nucleic acids generated during ICH are all TLRs ligands (Lin et al., 2012; Fang et al., 2013; Zhou et al., 2014; Wan et al., 2016; Fu et al., 2021). After binding to these ligands, TLRs signaling is activated. TLR4 simultaneously activates two parallel downstream pathways of myeloid differentiation factor 88 (MyD88) and TIR-domain-containing adapter-inducing interferon-β (TRIF) while TLR2 recruits only MyD88. Both of them lead to the activation of transcription factor NF-κB (Sansing et al., 2011; Wang Y.-C. et al., 2013; Fei et al., 2019). NF-κB is a crucial signal for microglial M1-polarization and inflammatory factors expression. During the process, inhibitors of NF-κB kinase (IKK) are activated firstly, which cause the phosphorylation and degradation of NF-κB inhibitor (Iκb; Fei et al., 2019). After that, NF-κB dimer is released and enters the nucleus to regulate transcription for M1-polarization. Of note, NF- κB can be detected in the peripheral circulation, which is a biomarker to determine the severity of brain damage.

#### MAPK

Mitogen-activated protein kinase (MAPK) is a member of the serine/threonine kinase family, which includes P38, Extracellular Signal-Regulated Kinase1/2 (ERK1/2), c-Jun N-terminal kinase (JNK) pathways (Sun and Nan, 2016). MAPK not only enters the nucleus to regulate the transcription processes but also increases the activity of NF-κB in the cytoplasm (Wei et al., 2019). After ICH, MAPK is activated by inflammatory factors, thrombin, and glucocorticoid, MAPK signaling plays a critical role in microglia survival and M1-polarization.

#### NLRP3

Inflammasome NLR Family, Pyrin domain containing protein 3 (NLRP3) is a kind of intracellular multi-molecular protein complex that is involved in inflammation (Walsh et al., 2014; Luo et al., 2019). NLRP3 activates lyase caspase-1, an enzyme that trims microglia secreted pre-IL-1β and pre-IL-18 into mature IL-1β and IL-18 (Ren et al., 2018), which makes NLRP3 a promising target of inflammatory regulation. In the mouse ICH model, intraventricular injection of NLRP3 siRNA immediately reduced inflammatory response and brain damage.

#### PPAR-γ and Nrf2

Peroxisome proliferator-activated receptor (PPAR-γ) and Nuclear erythroid 2 related factor 2 (Nrf2) are important signals of M2-polarization (Zhao X.-R. et al., 2015). Nrf2 is a basic leucine zipper (bZIP) protein that enters the nucleus to regulate transcription. PPAR-γ is a highly-expressed nuclear hormone receptor in microglia. PPAR-γ and Nrf2 actually work together with overlapping functions. They enhance the expression of Arg-1, IL-4, and CD36, which enables microglia in phagocytosis and tissue repair (Xia et al., 2015; Wang J. et al., 2018). Except for that, PPAR-γ and Nrf2 jointly regulate the expression of hundreds of antioxidant genes including heme oxygenase-1 (HO-1; Culman et al., 2007; Shang et al., 2013).

#### STATs

As a common transcription signal for cytokines, signal transducer and activator of transcriptions (STATs) family exert their effect on both M1 and M2 polarization (Tschoe et al., 2020). Microglia express a large number of cytokine receptors, such as IL-1R, TNFR, IL-4R, which activate the downstream Janus kinase (JAK)-STATs signal. Among STATs, STAT1 promotes M1 polarization and inflammatory factors expression (Bai et al., 2020). STAT4/6 promotes M2 polarization and the expression of Ym-1 and Fizz1 (Righy et al., 2016). Intriguingly, STAT3 was demonstrated to be involved in both M1/M2 polarization (Hu et al., 2015).

### M1-M2 Phenotypic Transformation

It is observed that single microglia express both M1/M2 phenotypic markers (Ransohoff, 2016; Tschoe et al., 2020). Neither M1 nor M2 should be considered as a microglial final differentiation form. The ability of microglia to switch between M1/M2 phenotypes is always a fascinating topic. However, the mechanism for this phenotypic transformation is really elusive. M1 and M2 microglia not only perform distinct cellular functions but also have incompatible polarization processes. For example, *in vitro*, PPAR-γ significantly inhibits the activation of NF-κB and STAT1/3 (Fang et al., 2014). In like manner, inflammatory cytokines and TLRs inhibit microglia in CD36 expression (Zhou et al., 2014; Yuan et al., 2015).

Recently, the relationship between microglia phenotype and metabolic status has attracted much attention. Microglia in different phenotypes show different oxidative metabolism (Eun Jung et al., 2020). Compared to M1 microglia, M2 microglia have significantly lower oxygen consumption (Orihuela et al., 2016). Therefore, it has been speculated that intracellular stress environment and energy crisis promote M2 polarization by influencing mitochondrial metabolism. The reactive oxygen species (ROS) released by M1 microglia has been found to activate Nrf2, which contributes to microglial M2 polarization (Duan et al., 2016; Hu et al., 2016; Qu et al., 2016). In addition, Adenosine 5‘-monophosphate activated protein kinase (AMPK), as a key molecule regulating bioenergy metabolism, is activated under cellular energy crisis and oxidative stress (Saikia and Joseph, 2021). Evidence indicates that AMPK contributes to Nrf2 activation as well (Zhao et al., 2018; Zheng et al., 2019). In other words, the initiative activation of M2 microglia may be a type of self-protection when M1 phenotype creates an immoderate oxidative stress (Barakat and Redzic, 2015). More in-depth research in the mechanism of microglial phenotypic transformation may provide insights into innovative therapeutic strategies for ICH.

### Preclinical Researches Targeting Microglia

In view of the serious inflammatory brain injury, whole microglia population deletion by knocking out microglial survival signal receptor colony-stimulating factor 1 receptor (CSF1R) achieved an early therapeutic effect in rodent experiments (Li et al., 2017; Shi et al., 2019). Of course, increasing the M2/M1 phenotypic proportion of microglia usually brings more satisfactory results (Wang J. et al., 2018; Bai et al., 2020), and it has become the most frequently studied therapeutic method. Relying on the aforementioned targets, experimental therapeutic studies on the precise regulation on microglia phenotype are developing rapidly, and relevant drugs are summarized in Table 3 (Hu et al., 2011; Ohnishi et al., 2013, 2019; Yang et al., 2014a,b, 2018; Iniaghe et al., 2015; Zhao et al., 2015a,b, 2018; Flores et al., 2016; Shi et al., 2016; Sukumari-Ramesh and Alleyne, 2016; Zhang et al., 2016; Anan et al., 2017; Chen-Roetling and Regan, 2017; Lan et al., 2017a; Wang J. et al., 2017; Wei et al., 2017; Xu et al., 2017; Zeng et al., 2017; Chen C. et al., 2018; Chen S. et al., 2018; Fu et al., 2018; Han et al., 2018; Li X. et al., 2018; Qiao et al., 2018; Ren et al., 2018; Wang et al., 2018b; Liang et al., 2019; Song and Zhang, 2019; Xi et al., 2019; Zhou F. et al., 2019; Cheng et al., 2020; Ding et al., 2020).

**Table 3:** Preclinical researches on microglial regulation for ICH therapy.

Drugs	Targets	Species/Models	Results
Ginkgolide B	TLR4	rats/autologous blood	reduce inflammatory cytokine, lessen neuronal cell apoptosis.
Ligustilide	TLR4	mice/autologous blood	reduce inflammatory cytokine, induced neurological deficits.
Magnolol	TLR4	rats/collagenase	reduce the brain water content, attenuated neurological deficits.
Pinocembrin	NF-κB	mice/collagenase	reduce lesion volume and neurologic deficits.
Sparstolonin B	NF-κB	mice/autologous blood	reduce inflammatory cytokine and brain edema.
Curcumin	NF-κB	mice/autologous blood	inhibit inflammation and neurological impairment.
Protocatechuic acid	NF-κB	mice/collagenase	inhibit oxidative stress, inflammation and apoptosis.
Annexin A1	MAPK	mice/collagenase	attenuate brain edema, improved short-term neurological function.
Sesamin	MAPK	rats/collagenase	suppress microglial activation, prevent neuron loss.
Fisetin	NF-κB	mice/collagenase	reduce inflammatory cytokine, brain edema and cell apoptosis.
Theaflavin	NF-κB	rats/collagenase	alleviate the behavioral defects, inhibit the neuron loss and apoptosis.
fimasartan	NLRP3	rats/collagenase	attenuate brain edema and improve neurological functions.
dexmedetomidine	NLRP3	mice/autologous blood	reduce inflammatory cytokine, improve neurological function.
AC-YVAD-CMK	NLRP3	mice and rats/collagenase	reduce brain edema and improve neurological function.
MCC950	NLRP3	mice/autologous blood and collagenase	attenuate neuro-deficits and perihematomal brain edema.
Dimethyl fumarate	Nrf2	mice and rats/collagenase and autologous blood	improve neurological deficits.
Nicotinamide mononucleotide	Nrf2	mice/collagenase	suppress neuroinflammation and oxidative stress.
Shogaol	Nrf2	mice/collagenase	suppress oxidative stress and improve neurological function.
sulforaphane	Nrf2	mice and rats/ autologous blood	improve hematoma clearance.
Tert-butylhydroquinone	Nrf2	mice/collagenase	suppress oxidative stress and improve neurological function.
Isoliquiritigenin	Nrf2	rats/collagenase	alleviate neurological deficits.
Andrographolide		rats/autologous blood	alleviate neurobehavioral disorders and brain edema.
monascin	Nrf2	rats/collagenase	improve neurological deficits.
Sinomenine	Nrf2	mice/autologous blood	improve neurological deficits.

## Clinical Researches Targeting Microglia

Translational research in medication development has never been effortless. Although many preclinical researches have got positive results in ICH treatment, large clinical trials on microglia intervention are second to none. Conservatively, the therapeutic effect of minocycline, deferoxamine, fingolimod, thiazolidinediones (TZDs), and statins are relatively promising. Related clinical researches have been briefly summarized in Table 4.

**Table 4:** Clinical researches on microglial regulation for ICH therapy.

Drugs	Continent	No. of patients	Outcomes	Efficacy	References
minocycline	North America	10	NIHSS, mRS, mortality	NO	Chang et al. (2017)
	North America	8	mRS	NO	Fouda et al. (2017)
deferoxamine mesylate	Asian	47	hematoma volume, edema	YES	Yu et al. (2017)
fingolimod	Asian	23	hematoma volume, NIHSS	YES	Fu et al. (2014)
	Asian	11	edema	YES	Li et al. (2015)
statins	Europe	29	NIHSS, mortality	YES	Tapia-Pérez et al. (2013)
	North America	38	hematoma volume; edema	YES	Witsch et al. (2019)

*NIHSS, National Institute of Health stroke scale; mRS, modified Rankin Scale*.

Minocycline is an ordinary broad-spectrum antibiotic. It could pass through the blood-brain barrier freely and has a neuronal protection effect (Yang et al., 2019). With pleiotropic properties, minocycline scavenges free radical and promotes M1-M2 phenotypic transformation of microglia in piglet and rodent ICH models (Möller et al., 2016; Dai et al., 2019; Wang G. et al., 2019). When applied in ICH clinical trials, minocycline has not been demonstrated to produce favorable outcomes on 3-month functional independence and behavior score, but significantly depresses the levels of circulating inflammatory components (Fouda et al., 2017; Malhotra et al., 2018). It may be due to the fact that oral administration does not produce sufficient potency concentrations in brain parenchyma.

Deferoxamine is a classical iron-chelating agent. Except for reducing oxidative damage, it effectively reinforces the function of M2 microglia (Hu et al., 2019). RBC CD47 is a signal that stops itself from being swallowed by microglia (Song et al., 2021; Ye et al., 2021). Deferoxamine inhibited CD47 expression on RBCs and accelerated hematoma absorption conspicuously in pig models (Cao et al., 2016; Hu et al., 2019). In patients with spontaneous ICH, consecutive administration of deferoxamine mesylate for 5 days significantly reduces hematoma volume and brain edema progression (Yu et al., 2017).

Fingolimod is an S1PR agonist previously used for multiple sclerosis, which can directly activate M2 microglia. In ICH preclinical experiments, fingolimod has been demonstrated to inhibit brain edema and reduce the numbers of apoptotic cells (Rolland et al., 2013; Lu et al., 2014; Sun et al., 2016). When applied to clinical trials, 3 days consecutive oral administration of fingolimod shows beneficial effects on decreasing the numbers of lymphocytes and NK cells in circulation, controlling perihematomal brain edema (PHE), and ameliorating neurological deficits (Fu et al., 2014; Li Y.-J. et al., 2015).

TZDs, including pioglitazone and rosiglitazone, have a function in activating M2 microglia as PPAR-γ agonist (Song et al., 2018). In the rodent model, intraperitoneal injection of rosiglitazone increases the expression of CD36 on microglia, promotes hematoma clearance, and inhibits inflammatory factors expression (Chang C.-F. et al., 2017; Mu et al., 2017). TZDs have long been designed for clinical trials (Gonzales et al., 2013), but have not yet shown significant results.

Statins (HMG-CoA reductase inhibitors) are widely prescribed medications for the management of hypercholesterolemia. The potential of Statins for ICH treatment has been revealed recently (Chen Q. et al., 2019). Mechanistically, Statins regulate microglial phenotype by inhibiting inflammatory signals and enhancing PPAR-γ activity (Wang et al., 2018a; Bagheri et al., 2020). Although stains have been doubted for the safety of ICH treatment, they are ultimately deemed applicable in promoting neurological rehabilitation (Ribe et al., 2019). It has been demonstrated that statins improve the neurological function of ICH patients and reduce the mortality at 6 months (Tapia-Pérez et al., 2013; Witsch et al., 2019).

## Perspective

### The Balance of Yin and Yang

Though how to regulate microglia to promote brain recovery remains worth pondering in some sense, there are latent misgivings that excessive inhibition of M1 microglia and promotion of M2 microglia may turn into adverse effects in ICH treatment, where we should keep watchful eyes.

On the one hand, the immunoreactive materials secreted from M1 microglia appear to have delayed beneficial effects on brain repair. Solid evidence indicates that MMPs are necessary for angiogenesis, myelin remodeling, and axonal regeneration in ICH later stage (Lei et al., 2015; Fields, 2019). As well, infiltrating neutrophils and monocytes have been found conducive to hematoma clearance and inflammation regression (Lambertsen et al., 2019). Besides, the over-suppressed inflammatory status may increase brain infection risk since the systemic immunity also decreases after ICH (Saand et al., 2019).

On the other hand, the early organizational disruption may build the basis of neogenesis. M1 microglia destroy dying and defunct neurons in pieces, which lends a convenience for M2 phagocytosis (Hu et al., 2015). In addition, the deconstruction of dense tissue matrix made by M1 microglia provides space for the migration of neural precursors and synaptic remodeling (Lei C. et al., 2013). Also, M1 microglia impair BBB integrity, which is in favor of the hematoma clearance by free diffusion, especially when microglial phagocytic receptors are of inefficiencies in the ICH early phase (Righy et al., 2016, 2018).

As for M2 microglia, superfluous and prolonged existing growth factors will predictably cause abnormal tissue repair. Overexpression of Arg1 has been found to cause tissue scarring and brain dysfunction (Hesse et al., 2001), and excessive polyamines extraordinarily promote inflammatory response (Dudvarski Stankovic et al., 2016). Resting microglia plays a special role in tissue repair and remodeling (Cherry et al., 2014), and M2-M0 may be a necessary functional transformation after ICH.

In summary, it is really improper to consider that M1 and M2 microglial phenotypes are thoroughly opposite. Instead, their interaction, cooperation, and even codependency are waiting to be explored in the future. A balance of M1 and M2 microglial, rather than extremely choosing M2 over M1, ought to be achieved for ICH individualized treatment, just like the balance of yin and yang.

### Targeting Strategy

Although drugs with the pleiotropic ability of immune regulation may bring more benefits, not a few medical experiments failed just because of uncontrolled side effects. It is a neglected consensus that many microglial receptors and signaling molecules are meanwhile expressed or activated in other brain cells, such as astrocytes, oligodendrocytes, endothelial cells, and neurons. It is unwise to judge the holistic functions of concerned targets in the brain by taking only microglia into account. For example, CD163 helps microglia engulf and break down hemoglobin, whereas, inhibition of CD163 in the ICH acute phase unexpectedly reduces brain damage, possibly because inhibition of CD163 expressed on neurons decreases the Hb neurotoxicity induced neuronal death (Righy et al., 2018).

Hence, we need a kind of drug that has a high targeting specificity to microglia. Preferably, it’s expected to have sufficient liposoluble ability to pass through BBB and concentrate on microglia. Furthermore, it’s recommended to conjunctive use advanced medical technology such as intranasal administration, nanomaterials, and genetic technologies to achieve better intervention results for ICH treatment.

## Author Contributions

RB and ZF wrote and revised the manuscript. MY helped with the literature search and correction of the manuscript. BH and QH provided the conception and design of the review, and directed the writing of the manuscript. All authors contributed to the article and approved the submitted version.

## Conflict of Interest

The authors declare that the research was conducted in the absence of any commercial or financial relationships that could be construed as a potential conflict of interest.

## Publisher’s Note

All claims expressed in this article are solely those of the authors and do not necessarily represent those of their affiliated organizations, or those of the publisher, the editors and the reviewers. Any product that may be evaluated in this article, or claim that may be made by its manufacturer, is not guaranteed or endorsed by the publisher.
